# Phubbing and Caring Behaviors Among Emergency Nurses: The Mediating Role of Emotional Exhaustion

**DOI:** 10.1155/jonm/3017641

**Published:** 2026-10-26

**Authors:** Tao Lin, Qing Ye, Yongli Gao

**Affiliations:** ^1^ Emergency Department of West China Hospital Sichuan University/West China School of Nursing Sichuan University Chengdu China scu.edu.cn; ^2^ Institute of Disaster Medicine Sichuan University Chengdu China scu.edu.cn; ^3^ Nursing Key Laboratory of Sichuan Province Chengdu China scu.edu.cn

**Keywords:** caring behaviors, emergency nurses, emotional exhaustion, job demands-resources model, nursing management, phubbing

## Abstract

**Background:**

Phubbing (phone snubbing) in high‐pressure emergency department (ED) settings is associated with nurse–patient interaction challenges and patient safety concerns; however, its underlying process remains unclear.

**Aim:**

Grounded in the job demands‐resources model, in this study, we explored the relationship between phubbing and caring behaviors among ED nurses, tested the mediating role of emotional exhaustion, and identified potential intervention targets to inform nursing management practice.

**Design:**

A multicenter cross‐sectional study.

**Methods:**

Between March and June 2025, 512 ED nurses from 18 hospitals across five geographic‐economic tiers in Sichuan Province, China, were recruited via stratified multistage cluster sampling. Data were collected using the Chinese versions of the generic scale of phubbing (GSP), caring behaviors scale‐24 (CBS‐24), Maslach Burnout Inventory‐General Survey (MBI‐GS), and a demographic questionnaire. Common method bias was assessed using Harman’s single‐factor test and the marker‐variable technique. Hierarchical regression and bootstrap methods were used to test the mediating effect of emotional exhaustion.

**Results:**

The mean GSP, CBS‐24, and emotional exhaustion scores were 4.28 ± 0.88, 4.52 ± 0.46, and 3.18 ± 0.82, respectively. Hierarchical regression showed that, after controlling for demographics, phubbing was negatively associated with caring behaviors (*β* = −0.203, *p* < 0.001), explaining an additional 5.2% of variance. After emotional exhaustion was added, the direct association of phubbing decreased to *β* = −0.138 (*p* = 0.002). Emotional exhaustion was negatively associated with caring behaviors (*β* = −0.267, *p* < 0.001), explaining an additional 6.8% of variance. Bootstrap analysis indicated that emotional exhaustion partially mediated the relationship (indirect effect = −0.089, 95% CI [−0.126, −0.056]), accounting for 43.8% of the total effect.

**Conclusions:**

Phubbing is significantly associated with lower caring behaviors among ED nurses and is indirectly associated with lower caring behaviors through emotional exhaustion. Emotional exhaustion is a potential intervening node for nursing managers.

## 1. Introduction

Emergency nursing, characterized by high timeliness and uncertainty, requires nurses to maintain a sustained cognitive focus and emotional investment during triage assessment, critical care resuscitation, and dynamic monitoring [1–3]. However, the widespread use of smartphones is reshaping clinical work settings and has given rise to a novel form of workplace interactional deviance known as phubbing. The term, a portmanteau of “phone” and “snubbing,” refers to the act of ignoring someone in one’s physical presence by focusing on the mobile phone [4]. The International Patient Safety Goals emphasize effective communication and reduction of medical errors, and digital distraction behaviors are an emerging threat to these objectives [5]. Digital distraction among healthcare professionals is significantly associated with medication errors, patient falls, and communication‐related adverse events, suggesting that phubbing is not merely an individual disciplinary issue but also a managerial concern that affects patient safety and organizational performance [6]. In China, smartphones are deeply integrated into clinical communication, with WeChat—the dominant multipurpose messaging platform—widely used for clinical work coordination; this blurs the boundary between work‐related and personal use and may exacerbate phubbing in EDs.

Caring behaviors are a core indicator of nursing service quality and refer to the sum of nurses’ explicit behaviors across multiple dimensions, including professional technical competence, humanistic care delivery, patient dignity maintenance, and emotional connection building [7, 8]. The Caring Behaviors Inventory was based on Watson’s theory of human caring and was later refined by Wu et al. into the 24‐item caring behaviors scale (CBS‐24) [7]. This instrument has been widely used across cultural contexts, and its four dimensions—assurance, knowledge and skills, respectfulness, and commitment—form the foundation of patients’ perceived quality of care [7]. In the context of emergency management, high‐quality caring behaviors manifest not only in the precision of technical operations but also in psychological reassurance for patients with anxiety and informational support for their families [9]. Existing research on caring behaviors has focused primarily on individual characteristics or organizational factors, with limited attention given to the potential erosion of caring behaviors due to emerging digital‐age workplace behaviors [10].

Although the psychosocial consequences of phubbing in the general population have been widely discussed [10, 11], empirical research in nursing remains sparse. Specifically, three gaps exist: first, studies have mainly included university students or general workplace populations as research participants, with insufficient attention to emergency nurses, a group exposed to high stress and high attentional demands. Second, phubbing has been treated as an isolated individual habit rather than examined within a theoretical framework of the work context, leaving unanswered questions about why it occurs and how it translates to nursing outcomes. Third, the lack of examination of mediating mechanisms leaves nursing managers without precise intervention targets—should they regulate the behavior itself or eliminate the internal psychological states driving the behavior?

The job demands‐resources (JD‐R) model provides a systematic theoretical lens to address these gaps [12, 13]. Proposed by Demerouti et al. and increasingly applied in nursing management research, the model posits that job characteristics can be classified as job demands and job resources. Job demands refer to the physical, psychological, social, or organizational aspects of a job that require sustained physical or mental effort (e.g., high‐pressure emergency work, emotional labor, and night‐shift load). Job resources refer to functional means that help achieve work goals, mitigate job demands, or stimulate personal growth (e.g., specialist competence, social support, and autonomy). The JD‐R model states that when job demands are excessively high and job resources are insufficient, individuals undergo a resource depletion process (e.g., emotional exhaustion), which subsequently impairs work engagement and task performance. Concurrently, individuals may initiate resource recovery strategies, although not all are adaptive [14].

When applying the JD‐R model to this study, the following logic chain can be constructed: emergency nursing, as a high‐demand environment, continuously depletes nurses’ emotional and cognitive resources, resulting in emotional exhaustion. When organizational job resources (e.g., rest opportunities, professional support, and decision‐making autonomy) are insufficient to compensate for this depletion, nurses may resort to maladaptive resource‐recovery strategies, such as seeking immediate emotional relief through virtual smartphone interactions, which manifest as increased phubbing. However, this strategy fails to genuinely restore resources. Instead, it consumes scarce cognitive resources, disrupts work continuity, and triggers interpersonal conflicts with colleagues and patients, resulting in further resource loss and ultimately a decline in the quality of caring behaviors. From a nursing management perspective, identifying whether phubbing is merely an individual behavioral issue or a symptom of systemic resource depletion is important for designing targeted organizational interventions rather than simplistic punitive policies. Accordingly, this study proposes the following hypothesis (Figure 1):

**FIGURE 1:**
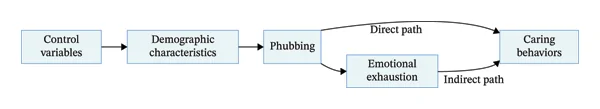
Conceptual framework based on the job demands‐resources model. *Note.* Arrows indicate hypothesized associations, not causal directions. H1: phubbing is negatively associated with caring behaviors. H2: emotional exhaustion mediates the association between phubbing and caring behaviors. Control variables: sex, age, years of ED experience, specialist nurse certificate, professional title, hospital grade, monthly income, and average monthly night shifts.

By testing these hypotheses, we aimed to identify evidence‐based intervention strategies for emergency nursing managers that transcend simplistic “phone bans,” improving the quality of nursing care through the dual approaches of optimizing work resources and managing emotional exhaustion. To the best of our knowledge, this study is one of the first to specifically examine phubbing—as distinct from general smartphone distraction—within the JD‐R framework in emergency nursing to systematically examine the relationship between phubbing, emotional exhaustion, and caring behaviors in the context of emergency nursing in China.

## 2. Materials and Methods

### 2.1. Study Design, Setting, and Sampling

This study employed a multicenter cross‐sectional correlational design and was reported in accordance with the Strengthening the Reporting of Observational Studies in Epidemiology guidelines [15]. A stratified multistage cluster sampling method was used: In Stage 1, the study area was divided into five geographic‐economic tiers in Sichuan Province: Chengdu Plain, Southern Sichuan, Northeastern Sichuan, Northwestern Sichuan, and Panxi. In Stage 2, within each tier, hospitals were stratified by grade (tertiary A, tertiary B, and secondary A), and 18 hospitals (8 tertiary and 10 secondary) were selected using a random number table. In Stage 3, the emergency department (ED) of each selected hospital was treated as a cluster unit, and all eligible on‐duty nurses were included.

### 2.2. Participants

The inclusion criteria were as follows: (1) holding a valid nursing practice certificate issued by the People’s Republic of China, (2) having worked continuously in the ED for ≥ 6 months, (3) being on duty during the survey period, and (4) providing informed consent and voluntary participation.

The exclusion criteria were as follows: (1) trainees, rotating, or intern nurses; (2) those on sick, maternity, or personal leave for > 2 weeks during the survey period; (3) those diagnosed with a mental disorder and currently taking psychiatric medication; (4) those who had experienced a major medical dispute within the past 3 months and were in a state of psychological stress; and (5) those simultaneously holding administrative positions and spending > 2 days per week away from clinical frontline duties.

Sample size calculation: based on Cohen’s [16] effect size estimates, a medium effect size of *f* ^2^ = 0.15 was set, with a significance level of *α* = 0.05 (two‐tailed), power of 1−*β* = 0.95, and number of predictors *k* = 16. The minimum required sample size, calculated using G∗Power 3.1.9.7 [17], was 208. Considering the design effect of the multistage cluster sampling (Deff = 1.5, estimated from an average cluster size of approximately 28 nurses per hospital and an intraclass correlation coefficient of 0.05) [18] and an anticipated 20% invalid response rate, the final required sample size was at least 374. In total, 632 questionnaires were distributed, and 512 valid questionnaires were obtained, yielding an effective response rate of 81.0% (Figure 2).

**FIGURE 2:**
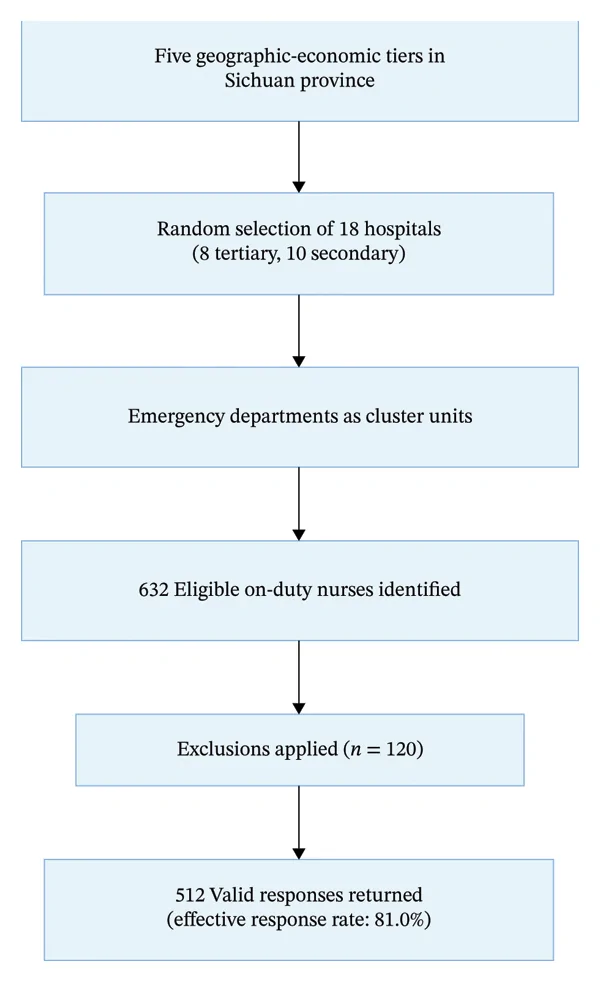
Sampling flowchart.

### 2.3. Instruments

General Information Questionnaire: this is a self‐designed questionnaire, comprising 11 demographic and occupational variables, including sex, age, education, professional title, years of ED experience, employment type, possession of an emergency specialist nurse certificate, hospital grade, marital status, average monthly night shifts, and monthly income. Professional titles were classified into four categories based on actual clinical appointment status: nurse, primary nurse, nurse practitioner, and associate/chief nurse. In China’s nursing title system, “nurse practitioner” (zhuguan hushi) refers to an intermediate clinical title, distinct from the advanced practice nurse role in Western contexts. Tertiary A is the highest hospital accreditation level in China.

Chinese version of the generic scale of phubbing (GSP): the Chinese version was translated by Zhang et al. [19]. The scale contains 15 items across four dimensions: nomophobia (4 items), interpersonal conflict (4 items), self‐isolation (4 items), and problem recognition (3 items). Items are rated on a 7‐point Likert scale (1 = never, 7 = always) with no reverse scoring. The total scores ranged from 15 to 105, with higher mean scores indicating more severe phubbing. Cronbach’s *α* of the Chinese version was 0.91; in this study, the total scale *α* was 0.91, and subscale *α* ranged from 0.78 to 0.86.

Workplace applicability: as a generic scale, GSP items (e.g., “I feel anxious when my phone is not nearby,” “I ignore people around me because of my phone use”) are not restricted to specific contexts, enabling the capture of nurses’ phubbing tendencies in clinical work settings. In this study, instructions explicitly asked nurses to respond “based on your actual work situation,” contextualizing the measurement. However, we acknowledge that this scale does not distinguish work‐related from personal smartphone use—a significant limitation. Future research should employ situation‐specific scales or supplement with objective usage data (e.g., screen time records).

Chinese version of the CBS‐24: the Chinese version was translated by Da et al. [20]. The scale contains 24 items across four dimensions: assurance (8 items), knowledge and skills (5 items), respectfulness (6 items), and commitment (5 items). Items are rated on a 6‐point Likert scale (1 = *never*, 6 = *always*) to calculate the mean score. Scores ranged from 1 to 6, with higher scores indicating a better quality of caring behavior. Cronbach’s *α* of the Chinese version was 0.96; in this study, the total scale *α* was 0.95, and subscale *α* ranged from 0.82 to 0.91.

Chinese version of the Maslach Burnout Inventory‐General Survey (MBI‐GS): the Chinese version translated by Li et al. was adopted [21]. This scale contains 16 items across three dimensions: emotional exhaustion (5 items), depersonalization (4 items), and personal accomplishment (7 items). Items were rated on a 5‐point Likert scale (1 = never, 5 = always), with personal accomplishment reverse‐scored. This study uses emotional exhaustion as a core mediating variable. Cronbach’s *α* of the Chinese version ranged from 0.75 to 0.88 across dimensions; in this study, the total scale *α* was 0.88, and the emotional exhaustion dimension *α* was 0.90.

### 2.4. Data Collection Procedure and Quality Control

From March 2025 to June 2025, the researchers contacted the head nurses of the sampled EDs directly, explained the study purpose and completion requirements, and shared a Wenjuanxing (a widely used Chinese online survey platform similar to SurveyMonkey) electronic questionnaire link via WeChat. Data collection was distributed across four distinct time windows corresponding to seasonal shifts in ED patient volume: March (pre‐spring festival peak), April (post‐peak normalization), May (routine period), and June (early summer increase). Within each hospital, head nurses distributed the survey link at the beginning of each week and sent one reminder 24 h before the 48‐h deadline. Response time monitoring showed a median completion time of 12.3 min (interquartile range [IQR]: 8.5–18.7 min), with 23 questionnaires excluded for completion time < 5 min.

The head nurses organized eligible nurses in their departments to complete the survey within a relatively concentrated timeframe (typically 1 week). Nurses clicked on the link via WeChat to access the questionnaire page and submitted it automatically upon completion. Each WeChat account was limited to one submission, and logic jumps, mandatory item validation, and response time monitoring were enabled (< 5 min deemed invalid). For departments that did not complete the survey within 48 h, the researchers sent one reminder via WeChat.

WeChat was selected because it is the mainstream internal communication tool used in Chinese medical institutions. Organizing completion through department WeChat groups or the head nurse’s personal WeChat ensured high accessibility and ecological validity, leveraged the head nurse’s organizational authority to improve response rates, and guaranteed anonymity. No personally identifiable information was collected, and head nurses could only view the overall completion status of their department, not individual responses.

Voluntary participation safeguards: (1) an informed consent statement on the first page explicitly stated that “participation is entirely voluntary, and refusal or withdrawal will not result in any adverse consequences; ” (2) head nurses were responsible only for forwarding the link and organizing the timeframe and were prohibited from monitoring the completion process; (3) questionnaires were completed independently on nurses’ personal mobile phones, with automatic encrypted transmission to the research server; head nurses could not access individual responses; (4) a 48‐h response window was provided, allowing nurses to choose their preferred completion time.

Confidentiality safeguards: (1) no personally identifiable information was collected (name, employee ID, or specific department name); (2) IP addresses were used solely for identifying duplicate submissions and deleted before analysis; and (3) data storage employed double encryption, with access restricted to the first and corresponding authors.

Social desirability bias control: (1) Anonymous completion was used to reduce evaluation anxiety; (2) instructions emphasized that “there are no right or wrong answers; please respond based on your true feelings; ” (3) reverse‐scored items and attention check items were included (e.g., “please select “always” for this item”); (4) statistical controls included Harman’s single‐factor test and the marker‐variable technique (see Section 2.5).

### 2.5. Common Method Bias (CMB) Control and Assessment

Because self‐report scales were used to collect data at a single time point, CMB was a risk factor. Anonymity, different instructions and anchors for different scales, and reverse‐worded items were used. Statistically, Harman’s single‐factor test and the marker‐variable technique were used: unrotated principal component analysis of all scale items showed that the first factor explained 28.4% of variance (< 40% threshold). Using “number of nonprofessional books read per month” as a marker variable, zero‐order correlations between the marker variable and the three major study variables (GSP, CBS‐24, and emotional exhaustion) were all below 0.08 (*r* = 0.02–0.08, *p* > 0.05). After controlling for the marker variable, partial correlations between the major variables changed minimally (Δr = 0.01–0.04, all < 0.05), indicating that CMB did not significantly influence the results.

### 2.6. Ethical Considerations

This study was approved by the Ethics Committee of West China Hospital, Sichuan University (Approval No. 2024(1162); approval date: 18 June 2024). The ethical approval remained valid throughout the entire study period, and all data collection (March–June 2025) was conducted within the approved timeframe. Written permission was obtained from the nursing departments and hospital offices of all participating hospitals. The study adhered to the principles of the Declaration of Helsinki, and all participants signed informed consent forms.

### 2.7. Statistical Analysis

Missing data were handled by listwise deletion as the proportion of missing values was less than 5%; missing completely at random (MCAR) data were supported by Little’s MCAR test (*p* > 0.05). Data were analyzed using SPSS 26.0 (IBM Corp., Armonk, N.Y., USA) and the PROCESS macro v4.1. Continuous variables are presented as mean ± standard deviation (M ± SD) and categorical variables as frequency (*n*) and percentage (%). The Kolmogorov–Smirnov test was used to assess normality. Group comparisons were conducted using independent‐samples *t*‐tests and one‐way ANOVA, with Bonferroni post‐hoc tests and effect sizes reported as Cohen’s d or partial *η* ^2^. Pearson’s correlation analysis was used to examine variable associations. Hierarchical multiple regression was used to test the predictive effects of variables on caring behaviors; professional title was treated as a multicategorical variable with dummy coding (using “nurse” as the reference group). Cohen’s *f* ^2^ was calculated as an effect size indicator (0.02 = small, 0.15 = medium, 0.35 = large). Hayes’ PROCESS macro Model 4, using the bootstrap method (5000 re‐samples, 95% bias‐corrected confidence intervals), was used to test the mediating effect of emotional exhaustion. The significance level was set at *α* = 0.05. Multicollinearity was assessed using variance inflation factors (VIF) and tolerance values. All VIF values were below 3.0 (range: 1.2–2.8), and tolerance values were above 0.30, indicating no multicollinearity concerns.

Variable selection for multivariate models followed a two‐step procedure. First, all demographic and occupational variables were tested for bivariate associations with the main study variables (GSP and CBS‐24). Second, variables with *p* < 0.10 in bivariate analyses or with established theoretical relevance as confounders were retained as control variables in the hierarchical regression and mediation analyses [22, 23]. This included sex, age, years of ED experience, specialist nurse certificate, professional title, hospital grade, monthly income, and average monthly number of night shifts. Sex, age, and hospital grade were retained despite nonsignificant in bivariate analyses (*p* > 0.05) because they represent well‐established demographic and organizational confounders in nursing workforce research [24]. Marital status was excluded from the multivariate models because of its nonsignificant bivariate associations (*p* > 0.05) and lack of theoretical relevance as a confounder in this context.

## 3. Results

### 3.1. Characteristics of Emergency Nurses

Among the 512 emergency nurses, 426 were female (83.2%) and 86 were male (16.8%). Nurse practitioners constituted the largest professional title group (35.5%), and nearly three‐quarters of them had ≤ 10 years of ED experience (74.7%). Most worked in tertiary hospitals (57.8%) and reported a monthly night‐shift frequency of 7–10 shifts (49.8%). Approximately one‐third held an emergency specialist nurse certificate (35.5%). Table 1 presents the demographic and occupational characteristics of the sample.

**TABLE 1:** Comparisons of GSP and CBS‐24 scores by characteristics of emergency nurses (*n* = 512).

Variable	Category	*n* (%)	Phubbing (GSP total score) (M ± SD)	F/t	*p*	Effect size	Caring behaviors (CBS‐24 total score) (M ± SD)	F/t	*p*	Effect size
Sex	Female	426 (83.2)	4.30 ± 0.88	0.852	0.395	—	4.53 ± 0.45	0.725	0.469	—
	Male	86 (16.8)	4.18 ± 0.92				4.48 ± 0.48			

Age (years)	≤ 30	228 (44.5)	4.35 ± 0.86	1.245	0.289	0.02	4.48 ± 0.47	2.105	0.123	0.034
	31–40	204 (39.8)	4.25 ± 0.89				4.55 ± 0.45			
	> 40	80 (15.6)	4.18 ± 0.92				4.58 ± 0.44			

Marital status	Married	298 (58.2)	4.25 ± 0.89	0.652	0.522	—	4.54 ± 0.45	0.885	0.414	—
	Unmarried	198 (38.7)	4.31 ± 0.87				4.49 ± 0.47			
	Divorced/widowed	16 (3.1)	4.35 ± 0.92				4.44 ± 0.52			

Years of ED experience	≤ 5	204 (39.8)	4.52 ± 0.85	8.562	< 0.001	0.033	4.38 ± 0.48	7.245	< 0.001	0.028
	6–10	179 (34.9)	4.18 ± 0.88				4.58 ± 0.44			
	> 10	129 (25.2)	4.05 ± 0.90				4.65 ± 0.42			

Specialist certificate	Yes	182 (35.5)	3.95 ± 0.82	−4.852	< 0.001	0.588	4.72 ± 0.40	5.125	< 0.001	0.688
	No	330 (64.5)	4.46 ± 0.88				4.41 ± 0.48			

Professional title	Nurse	160 (31.2)	4.48 ± 0.82	3.568	0.014	0.021	4.35 ± 0.48	4.215	0.006	0.025
	Primary nurse	136 (26.6)	4.28 ± 0.85				4.52 ± 0.45			
	Nurse practitioner	182 (35.5)	4.18 ± 0.90				4.62 ± 0.43			
	Associate chief nurse or higher	34 (6.6)	4.12 ± 0.94				4.68 ± 0.41			

Hospital grade	Tertiary	296 (57.8)	4.25 ± 0.89	−0.785	0.433	—	4.54 ± 0.45	0.852	0.394	—
	Secondary	216 (42.2)	4.32 ± 0.87				4.49 ± 0.47			

Monthly income	< ¥5000	112 (21.9)	4.55 ± 0.82	4.215	0.015	0.017	4.38 ± 0.50	5.012	0.007	0.020
	¥5000–8000	276 (53.9)	4.25 ± 0.88				4.55 ± 0.44			
	> ¥8000	124 (24.2)	4.08 ± 0.91				4.68 ± 0.42			

Monthly night shifts	≤ 6	154 (30.1)	4.08 ± 0.85	5.682	0.004	0.022	4.68 ± 0.43	6.125	0.002	0.024
	7–10	255 (49.8)	4.28 ± 0.88				4.52 ± 0.45			
	> 10	103 (20.1)	4.58 ± 0.90				4.32 ± 0.48			

*Note:* Post hoc multiple comparisons (Bonferroni) showed that the > 10 years group differed significantly from the ≤ 5 years group for both GSP and CBS‐24 (*p* < 0.01), the > ¥8000 group differed significantly from the < ¥5000 group (*p* < 0.05), and the > 10 night shifts group differed significantly from the ≤ 6 night shifts group (*p* < 0.01). Post hoc comparisons for professional title: the nurse group differed significantly from the primary nurse, nurse practitioner, and associate chief nurse or higher groups for both GSP and CBS‐24 (*p* < 0.05). No significant differences were found between the primary nurse, nurse practitioner, and associate chief nurse or higher groups (*p* > 0.05). Effect sizes for independent‐samples *t*‐tests are reported as Cohen’s d; effect sizes for one‐way ANOVA are reported as partial *η* ^2^. All demographic variables were tested for bivariate associations. Variables with *p* < 0.10 or theoretical relevance as established confounders in nursing workforce research were included in multivariate models: sex, age, years of ED experience, specialist nurse certificate, professional title, hospital grade, monthly income, and average monthly night shifts. Although sex, age, and hospital grade did not reach statistical significance in bivariate analyses (*p* > 0.05), they were retained to minimize omitted variable bias. Marital status was included in the bivariate comparisons but excluded from multivariate models because of its nonsignificant associations with both GSP and CBS‐24 (*p* > 0.05) and lack of theoretical relevance as a confounder.

### 3.2. Descriptive Statistics and Reliability of Scales

As shown in Table 2, the total GSP score of emergency nurses was above the theoretical midpoint, indicating a moderately high level of phubbing, with the nomophobia dimension being the most prominent and the problem recognition dimension being relatively lower. The total CBS‐24 score was moderately high, with the knowledge and skills dimension scoring the highest, whereas the humanistic care dimensions (respectfulness and commitment) scored relatively low. The emotional exhaustion score on the MBI‐GS was moderate. Cronbach’s *α* coefficients for all scales were within acceptable ranges, indicating good reliability.

**TABLE 2:** Descriptive statistics, normality tests, and reliability coefficients of the scales (*n* = 512).

Scale/dimension	No. of items	M ± SD	Skewness	Kurtosis	K‐S test *p*	Cronbach’s *α*
*GSP*						
Nomophobia	4	4.82 ± 0.95	0.25	−0.18	0.085	0.86
Interpersonal conflict	4	4.12 ± 0.90	0.18	−0.22	0.112	0.82
Self‐isolation	4	4.25 ± 0.92	0.22	−0.15	0.092	0.84
Problem recognition	3	3.92 ± 0.88	0.15	−0.20	0.125	0.78
GSP total	15	4.28 ± 0.88	0.2	−0.25	0.095	0.91

*CBS-24*						
Assurance	8	4.58 ± 0.48	−0.35	0.12	0.065	0.88
Knowledge and skills	5	4.68 ± 0.44	−0.42	0.18	0.072	0.85
Respectfulness	6	4.38 ± 0.52	−0.28	0.08	0.088	0.91
Commitment	5	4.42 ± 0.54	−0.30	0.15	0.08	0.82
CBS‐24 total	24	4.52 ± 0.46	−0.32	0.12	0.075	0.95

*MBI-GS*						
Emotional exhaustion	5	3.18 ± 0.82	0.28	−0.15	0.09	0.9
Depersonalization	4	2.78 ± 0.78	0.35	−0.08	0.105	0.85
Personal accomplishment	7	3.22 ± 0.72	−0.25	0.1	0.115	0.88

*Note:* K‐S, Kolmogorov–Smirnov test; all variables are *p* > 0.05, indicating a normal distribution.

### 3.3. Comparisons of Scale Scores by Characteristics

The results of univariate analysis are shown in Table 1. Sex, age, marital status, and hospital grade showed no significant effects on phubbing (GSP total score) or caring behaviors (CBS‐24 total score). By contrast, years of ED experience, emergency specialist nurse certification, professional title, monthly income, and average monthly night shifts had significant effects on scores on both the GSP and CBS‐24. Overall, nurses with junior titles (nurses), low seniority, no specialist certification, high‐frequency night shifts, and low income had more severe phubbing tendencies and lower quality of caring behavior, whereas those with specialist certificates and senior titles demonstrated better caring behaviors and less severe phubbing tendencies. After separating the titles, nurses scored significantly higher on phubbing and significantly lower on caring behaviors than primary nurses, nurse practitioners, and associate chief nurses (*p* < 0.05). No significant differences were found between primary nurses and nurse practitioners. However, primary nurses performed better than nurses on either scale.

### 3.4. Correlation Analysis

Correlations between the major variables are presented in Table 3. The total GSP score was significantly negatively correlated with the total CBS‐24 score and significantly positively correlated with emotional exhaustion. The total CBS‐24 score was negatively correlated with emotional exhaustion and depersonalization and positively correlated with personal accomplishment. Additionally, emotional exhaustion was positively correlated with all GSP dimensions and negatively correlated with all CBS‐24 dimensions, providing preliminary support for subsequent mediation testing.

**TABLE 3:** Pearson correlation matrix of major variables (*n* = 512).

	1	2	3	4	5
1. GSP total	1				
2. CBS‐24 total	−0.298	1			
3. Emotional exhaustion	0.338	−0.325	1		
4. Depersonalization	0.228	−0.215	0.485	1	
5. Personal accomplishment	−0.185	0.258	−0.268	−0.312	1

*Note:* Correlation coefficients are Pearson’s r with 95% confidence intervals in brackets: GSP‐CBS‐24 [−0.382, −0.214]; GSP‐emotional exhaustion [0.253, 0.419]; and CBS‐24‐emotional exhaustion [−0.409, −0.239]. All correlations with absolute *r* > 0.12 are significant at *p* < 0.01 (two‐tailed).

### 3.5. Hierarchical Regression Analysis of Factors Influencing Caring Behaviors

Table 4 presents the results of hierarchical regression. After controlling for demographic variables, years of ED experience, emergency specialist nurse certification, and monthly income showed a positive association with caring behaviors, whereas average monthly night shifts showed a negative association with caring behaviors. Among the control variables, sex (*β* = 0.038, *p* = 0.347), age (*β* = 0.022, *p* = 0.632), and hospital grade (*β* = 0.012, *p* = 0.712) were not significantly associated with caring behaviors, indicating that these demographic characteristics did not confound the relationships of interest. Using nurses as the reference group, nurse practitioners (*β* = 0.168, *p* = 0.001) and associate chief nurse or higher (*β* = 0.195, *p* = 0.005) showed a positive association with caring behavior, whereas the difference between primary nurses and nurses was not significant (*β* = 0.092, *p* = 0.093). After entering the total GSP score, the model’s explanatory power increased significantly; phubbing showed a negative association with caring behaviors, consistent with H1. After including emotional exhaustion, the model’s explanatory power again increased significantly; emotional exhaustion showed an independent negative association with caring behaviors. The direct association of the GSP total score with caring behavior was, however, markedly attenuated, although still significant, suggesting that emotional exhaustion may play a partial mediating role.

**TABLE 4:** Hierarchical regression analysis of factors influencing caring behaviors (*n* = 512).

Variable	Model 1 (controls)	95% CI	*t*	Model 2 (+phubbing)	95% CI	*t*	Model 3 (+ emotional exhaustion)	95% CI	*t*
	β			β			β		
*Controls*									
Sex	0.042	[−0.042, 0.126]	0.985	0.04	[−0.044, 0.124]	0.94	0.038	[−0.046, 0.122]	0.89
Age	0.015	[−0.076, 0.106]	0.325	0.014	[−0.077, 0.105]	0.3	0.022	[−0.068, 0.112]	0.48
Years of ED experience	0.128	[0.039, 0.217]	2.825	0.12	[0.031, 0.209]	2.65	0.115	[0.026, 0.204]	2.54
Specialist nurse certificate	0.165	[0.081, 0.249]	3.852	0.158	[0.074, 0.242]	3.68	0.152	[0.068, 0.236]	3.55
Primary nurse (ref.: nurse)	0.092	[−0.015, 0.199]	1.685	0.088	[−0.019, 0.195]	1.61	0.082	[−0.025,0.189]	1.5
Nurse practitioner (ref.: nurse)	0.168	[0.072,0.264]	3.452	0.162	[0.066, 0.258]	3.32	0.155	[0.059, 0.251]	3.18
Associate chief nurse or higher (ref.: nurse)	0.195	[0.060, 0.330]	2.845	0.19	[0.055,0.325]	2.77	0.182	[0.047, 0.317]	2.66
Monthly income	0.112	[0.025, 0.199]	2.632	0.108	[0.021, 0.195]	2.44	0.105	[0.018, 0.192]	2.37
Average monthly night shifts	−0.095	[−0.181,‐0.009]	−2.185	−0.090	[−0.176, ‐0.004]	−2.070	−0.082	[−0.168, 0.004]	−1.880

*Predictors*									
GSP total (phubbing)	—	—	—	−0.203	[−0.285,‐0.121]	−4.850^∗∗∗^	−0.138	[−0.216,‐0.060]	−3.15^∗∗∗^
Emotional exhaustion	—	—	—	—	—	—	−0.267	[−0.356,‐0.178]	−5.92^∗∗∗^

*Model fit*									
*R* ^2^	0.158			0.21			0.278		
Δ*R* ^2^	0.158			0.052			0.068		
F/F change	*F*(9.502) = 10.85^∗∗∗^			ΔF(1.510) = 32.98^∗∗∗^			ΔF(1.500) = 47.09^∗∗∗^		
Cohen’s *f* ^2^	0.188			0.065			0.086		
Durbin–Watson	1.975			—			2.005		

*Note: β* = standardized regression coefficient; 95% CI = 95% confidence interval; ref. = reference group (professional title dummy coded with “nurse” as reference category). Model 1: *F* (9, 502) = 10.85, *p* < 0.001. Model 2: Δ*R* ^2^ corresponds to the entry for GSP total; F change values reflect incremental prediction at each step. Model 3: Δ*R* ^2^ corresponds to the entry for emotional exhaustion. Cohen’s *f* ^2^ effect size interpretation: 0.02 = small, 0.15 = medium, and 0.35 = large. All variance inflation factor (VIF) values were below 3.0 (range: 1.2–2.8), and tolerance values were above 0.30, indicating no multicollinearity concerns. Effect values in Table 5 are unstandardized coefficients from the PROCESS macro (Model 4), which are not directly comparable in magnitude to the standardized *β* coefficients reported in this table. Sex, age, and hospital grade were nonsignificant in bivariate analyses (Table 1) but were retained as control variables based on their established theoretical relevance as demographic and organizational confounders in nursing workforce research.

Abbreviation: GSP, generic scale of phubbing.

^∗∗∗^ *p* < 0.001.

### 3.6. Mediation Effect Test of Emotional Exhaustion

Table 5 presents the bootstrap mediation test results. Emotional exhaustion partially mediated the relationship between phubbing and caring behaviors; the 95% confidence interval for the indirect effect did not contain zero, and the mediation effect accounted for 43.8% of the total effect, consistent with H2.

**TABLE 5:** Bootstrap test results of the mediating effect of emotional exhaustion.

Effect path	Effect value	SE	95% CI	Proportion of effect (%)
Total effect (X ⟶ Y)	−0.227	0.042	[−0.285, −0.121]	100
Direct effect (X ⟶ Y)	−0.138	0.04	[−0.216, −0.060]	56.20
Indirect effect (X ⟶ M ⟶ Y)	−0.089	0.018	[−0.126, −0.056]	43.80

*Note:* X = GSP total (phubbing); Y = CBS‐24 total (caring behaviors); M = emotional exhaustion. Effect values are unstandardized coefficients from the PROCESS macro Model 4 (5000 bootstrap samples). Control variables are identical to those in the regression model (sex, age, years of ED experience, specialist nurse certificate, professional title, monthly income, average monthly night shifts). Standardized coefficients (*β*) from Table 4 and unstandardized coefficients from this table both indicate negative associations but are not directly comparable in magnitude.

## 4. Discussion

### 4.1. Overview of the Main Findings

This study found that (1) emergency nurses exhibit a moderately high level of phubbing, with nomophobia as the most prominent dimension; (2) overall caring behaviors are acceptable; however, the humanistic care dimensions (respectfulness and commitment) are relatively weak; and (3) phubbing is directly associated with lower caring behaviors and indirectly associated with lower quality of care through emotional exhaustion, with the mediation effect accounting for 43.8%. These findings provide an organizational‐behavioral explanation and an intervention pathway for emergency nursing management that transcends individual moral discipline.

Recent international research provides valuable insights into the antecedents and consequences of emotional exhaustion among emergency nurses. Köksal and Mert [25] found that courage and interactional justice are key psychological resources alleviating emotional exhaustion among Turkish emergency nurses, echoing our finding regarding specialist certificates as resource‐protective factors. Mert and Köksal [26] further confirmed that grit and social cooperation buffer the negative impact of risk perception on work performance, suggesting that the resource dimension in the JD‐R model should be extended to include psychological capital. Notably, Tufan et al. [27], in their study of information technology behaviors, found that both phubbing and being phubbed damage friendship satisfaction; this bidirectional interaction perspective suggests that future emergency nursing research should attend to triadic nurse‐patient‐colleague phubbing dynamics.

### 4.2. Phubbing as a Maladaptive Resource Recovery Strategy Within the JD‐R Framework

The JD‐R theory classifies job characteristics as demands and resources, positing that when job demands chronically exceed resource supply, employees enter a resource depletion pathway [12]. In this study, phubbing among emergency nurses can be viewed as a maladaptive attempt at resource recovery. Confronted with high emotional labor, high cognitive load, and unpredictable night‐shift rhythms in emergency work, nurses attempt to obtain transient emotional relief through immediate social feedback provided by smartphones. However, unlike genuine resource recovery activities, phubbing is essentially “pseudo‐recovery”—it consumes already scarce cognitive resources, disrupts work continuity, and may trigger interpersonal conflicts with colleagues and patients, thereby trapping nurses in a vicious cycle of “resource depletion ⟶ phubbing ⟶ further depletion.”

This interpretation aligns with the theoretical conclusions of Broetje et al. [28]’s integrative review on the JD‐R model: high job demands in nursing settings, when unsupported by adequate organizational resources, readily translate into emotional exhaustion and maladaptive coping behaviors. From a managerial perspective, simplistic behavioral bans may encounter strong resistance because smartphones have become a “psychological crutch” for some nurses in a state of resource depletion. If managers ignore investment in work resources and rely solely on regulatory constraints, they may drive phone use toward a more covert use, which paradoxically increases management difficulties.

### 4.3. Mediating Mechanism of Emotional Exhaustion: A Critical Node for Managerial Intervention

The core contribution of this study lies in verifying the partial mediating effect of emotional exhaustion (43.8%), indicating that nearly half of the negative effects can be prevented by interventions aimed at reducing emotional exhaustion. Within the JD‐R framework, emotional exhaustion is the central indicator of the resource depletion pathway, representing severe depletion of psychological energy. When emotional exhaustion was included in the model, the direct association between phubbing and caring behaviors, although still significant, was substantially reduced by 32.0%. This suggests that emotional exhaustion is an important intervening mechanism associated with the link between digital distraction and nursing quality [29].

This finding has direct strategic significance for nursing management; emotional exhaustion is a measurable, intervenable, organizational‐level variable. Compared with changing individual smartphone habits, reducing emotional exhaustion through optimized scheduling, increased staffing, and emotional labor compensation may be more managerially feasible and cost‐effective. As Dall’Ora et al. [30] observed in their systematic review, a robust association exists between nurses’ emotional exhaustion and patient safety incidents, and the amelioration of emotional exhaustion often requires structural organizational interventions rather than purely individual resilience training.

### 4.4. Interpretation Within the Chinese Cultural Context

The findings of this study should be understood within the specific cultural‐organizational context of China. According to Hofstede’s cultural dimensions theory, China exhibits high collectivism (score = 20) and high power distance (score = 80), characteristics that shape communication norms in EDs. On one hand, a collectivist culture emphasizes group harmony and face‐saving, making nurses sensitive to public criticism or conflict avoidance; smartphones may serve as a “safe harbor” for escaping interpersonal pressure. On the other hand, under high power distance, nurses may exhibit compliant responses to surveys organized by head nurses; although we employed anonymization measures, this cultural dynamic may have compressed the variance range of phubbing scores.

Furthermore, the deep integration of WeChat into Chinese medical institutions is distinctive. Unlike Western countries, which primarily rely on pagers or dedicated clinical information systems, WeChat groups in Chinese EDs simultaneously serve work coordination (handovers, critical value reporting) and social functions (departmental announcements, colleague interactions). This “public‐private hybrid” technological use ecology makes the demarcation of phubbing particularly difficult. Nursing managers must fully consider this technology‐cultural embeddedness when formulating smartphone use policies to avoid the disconnect between “one‐size‐fits‐all” bans and actual clinical needs.

### 4.5. Dimensional Differences in Caring Behaviors: Priority Sequence of Resource Conservation

CBS‐24 scores revealed a troubling imbalance: the “knowledge and skills” dimension performed best, whereas “respectfulness” and “commitment” lagged behind. This phenomenon can be reasonably explained by integrating the JD‐R theory and the conservation of resources theory: when nurses’ psychological resources are doubly depleted by emotional exhaustion and phubbing, individuals will prioritize allocating remaining resources to “core tasks” perceived as technical operations, whereas sacrificing “peripheral tasks” such as emotional labor [31]. In the emergency context, this “high‐technical, low‐humanistic” imbalance is particularly dangerous because ED patients often experience intense anxiety, fear, and uncertainty, and, therefore, the need for humanistic care is particularly urgent [9].

Nursing managers must be vigilant: an overemphasis on technical efficiency and throughput metrics may lead to a “dehumanizing” tendency in caring behaviors, and phubbing acts as an accelerator of this tendency [32]. The physical barrier of the smartphone screen not only cuts off eye contact between the nurse and patient but also transmits the implicit signal that “you are less important than my phone,” damaging patients’ trust in and satisfaction with the nursing profession.

### 4.6. Managerial Implications of Demographic Characteristics: Identifying High‐Risk Groups

Univariate analysis indicated that nurses with low seniority (≤ 5 years), junior professional titles (nurses), no emergency specialist certificates, high‐frequency night shifts (> 10/month), and low income exhibited more severe phubbing and weaker caring behaviors. This “four‐low” profile (low seniority, low title, low certificate, low income) constitutes the vulnerable “high‐demand, low‐resource” group predicted by the JD‐R model: nurses at the most junior title level lack emergency experience and psychological capital, making them more likely to resort to smartphone escapism under high pressure. By contrast, although primary nurses have a relatively short tenure, they possess some clinical experience and expectations for title promotion. Their resource endowment is superior to that of nurses; therefore, their phubbing and caring behavior performance falls between that of nurses and nurse practitioners. High‐frequency night shifts weaken cognitive control through sleep deprivation and circadian disruption, whereas low income reduces professional identity and organizational commitment, which may make nurses more prone to emotional detachment.

Notably, nurses who obtained emergency specialist certificates demonstrated significantly better caring behaviors and lower phubbing. Specialist training not only enhances technical competence but also substantively increases nurses’ job resource reserves—professional confidence, occupational identity, and decision‐making autonomy—thereby improving their capacity to adapt to high job demands [33]. This suggests that nursing managers should view specialist nurse training as an organizational‐level resource investment rather than merely a human capital expenditure.

### 4.7. Implications for Nursing Management

The following stratified management strategies are proposed based on the JD‐R model findings:

#### 4.7.1. Organizational Level: Work Resource Enhancement and Contextually Adaptive Smartphone Use Policies

The JD‐R theory clearly states that increasing job resources is the most effective way to offset the negative effects of high demand. The recommendations are as follows:

Specialist empowerment and tiered mentorship: expanding the coverage of emergency specialist nurse training, particularly by establishing specialist certification pathways in secondary hospitals and assigning senior specialist mentors to junior title nurses (including newly appointed nurses and low‐seniority primary nurses) to increase job resources through the dual pathways of clinical teaching and emotional support. Note that the resource needs of nurses and primary nurses differ. Nurses require focus on basic emergency skills and psychological safety establishment, whereas primary nurses need to focus on clinical reasoning and independent decision‐making capacity.

Contextually adaptive smartphone management framework: abandoning “one‐size‐fits‐all” bans and establishing a “red–yellow–green” tiered usage system based on emergency scene risk levels. Resuscitation rooms and critical care bays are red zones (personal phones are centrally stored before handovers, and only departmental emergency communication devices are retained). Triage desks and observation rooms are yellow zones (work‐related queries only, with “focus periods” established). The remaining areas are green zones. In the red zones, public departmental tablets or workstations are equipped to ensure alternative information access channels and reduce anxiety triggered by nomophobia [34].

#### 4.7.2. Leadership Level: Head Nurse Resource Provision and Early Identification of Emotional Exhaustion

Head nurses play a pivotal role in the JD‐R model as “resource allocators” and “buffers.” Early identification of emotional exhaustion: brief weekly meetings or handover intervals were used to identify signs of emotional exhaustion (e.g., irritability, frequent complaints, interaction avoidance, and frequent phone checking) through informal interviews and observation. Nurses with junior titles were the priority group for observation.

Microrecovery opportunity creation: adding 15–20 min “buffer periods” to shift scheduling, allowing nurses to engage in deep breathing, brief meditation, or free smartphone use in green recovery zones to prevent continuous emotional resource depletion. High‐quality microrecovery was found to effectively reduce emotional exhaustion and decrease maladaptive digital behaviors [35].

Role modeling and team norm shaping: head nurses should lead by keeping phones silent and storing them in fixed locations during resuscitation and ward rounds, thereby promoting a patient‐centered departmental culture through leadership behavior.

#### 4.7.3. Individual Level: Digital Literacy and Adaptive Resource Recovery Training

Incorporating “occupational digital literacy” into the mandatory continuing education curriculum for emergency nurses, with tiered training:

Cognitive restructuring and digital etiquette: using case discussions and role play to raise nurses’ awareness of the hidden damage phubbing causes to patient safety, nurse‐patient trust, and professional image, enhancing the cognitive level of the “problem recognition” dimension. Junior‐title nurses should receive focused training in nurse–patient communication scenario simulations.

Mindfulness‐based stress reduction (MBSR): providing an 8‐week mindfulness course for junior‐title and high‐frequency night‐shift nurses. Empirical studies have demonstrated that MBSR can effectively reduce emotional exhaustion, enhance attentional regulation, and provide a resource recovery strategy as an alternative to smartphone use [36].

Alternative coping strategies: teaching nurses to use grounding techniques (e.g., the “5‐4‐3‐2‐1” sensory technique) or brief physical disengagement (e.g., a 2‐min brisk walk in the corridor) to replace smartphone escapism during emotional high‐pressure moments, achieving adaptive replenishment of psychological resources.

#### 4.7.4. System Level: Human Resource Allocation and Emotional Labor Compensation

 Optimizing ED staffing and night‐shift models: implementing flexible scheduling based on patient flow fluctuations to reduce the average monthly night‐shift frequency per nurse; exploring “long night + consecutive rest” models to replace “frequent short nights” and ensuring sleep recovery. The night shift density of junior‐title nurses must not exceed the institutional average.
 Establishing an emotional labor compensation mechanism: incorporating emotional labor intensity into performance appraisal; providing special subsidies or priority promotion opportunities for nurses in high‐frequency night‐shift positions and high‐complaint‐risk posts, as well as junior‐title nurses who shoulder more foundational emotional labor. Such recognition can improve their sense of organizational fairness and resource gain [37].
 Based on the JD‐R framework, we propose a phased intervention roadmap for nursing managers: (a) immediate: implementing red‐zone phone storage in resuscitation areas; (b) short‐term: establishing microrecovery breaks (15–20 min) for junior nurses; (c) medium‐term: expanding emergency specialist certification as a job resource; (d) long‐term: restructuring night‐shift scheduling and formalizing emotional‐labor compensation.
 Emotional labor compensation: from punishment to support paradigm shift: The core managerial implication of this study is that phubbing governance should shift from “behavioral punishment” to “resource compensation.” Specific administrative mechanisms include
  1. Emotional labor point system: quantifying emotional labor intensity for high‐frequency night shift and high‐complaint‐risk positions into performance points, linked to professional title promotion and excellence evaluation;
  2. Mandatory recovery leave: for nurses screening positive for emotional exhaustion (MBI‐GS emotional exhaustion dimension ≥ 3.5), initiating a “restorative leave” program preserving full performance pay during leave, with replacement staffing provided;
  3. Peer support network: establishing structured “senior‐junior” pairing systems, with specialist nurses providing weekly one‐hour support sessions for nurses with ≤ 5 years of experience, covering clinical decision‐making coaching and emotional debriefing;
  4. Patient feedback loop: incorporating patient‐reported caring experience (e.g., patient version of CBS‐24) into departmental quality assessment, but using solely for system improvement rather than individual rewards/punishments, avoiding defensive care behaviors.

### 4.8. Limitations and Future Directions

This study has a few limitations. First, the cross‐sectional design precludes causal inferences. Reverse causality is plausible: emotionally exhausted nurses may seek smartphone escapism as a coping mechanism, or nurses with inherently lower caring tendencies may both phub more and report higher exhaustion. Future ecological momentary assessment (EMA) or longitudinal designs are warranted to disentangle these reciprocal dynamics. Second, self‐report scales may be subject to social desirability bias. Although this study controlled for this using Harman’s test and the marker‐variable technique, future studies should combine objective observation methods or patient‐reported evaluations of caring behaviors for triangulation. Third, this study did not distinguish between work‐related and personal smartphone use, which may have different mechanisms of influence on caring behaviors. Future research should use time‐use logs or app monitoring for finer differentiation. Fourth, although the sample covered hospitals at all levels in Sichuan Province, it was confined to a single province; future multicenter, cross‐regional comparative studies should examine the moderating effects of regional culture and healthcare system differences. Finally, this study tested only the single mediation of emotional exhaustion, and future research should incorporate work resources (e.g., social support and autonomy) as moderators to construct a moderated mediation model, providing more refined targets for managerial intervention.

#### 4.8.1. Limitation of Data Collection Method

Despite safeguards for voluntary participation, using head nurse‐organized WeChat group distribution may still involve some social desirability bias and perceived pressure. Future research should consider using independent third‐party platforms or combining patient‐reported outcome measures for triangulation.

#### 4.8.2. Variable Selection Strategy

The inclusion of control variables in multivariate models was guided by both statistical criteria (bivariate *p* < 0.10) and theoretical relevance as established confounders in nursing workforce research. Although sex, age, and hospital grade were not statistically significant in bivariate analyses, they were retained to minimize the risk of omitted variable bias, consistent with recommendations that control variables should be selected based on substantive and theoretical grounds rather than solely on bivariate statistical significance.

#### 4.8.3. Insufficient Contextual Specificity of Measurement Tools

The generic nature of the GSP is both a strength and a limitation. In Chinese emergency settings, smartphones are deeply integrated into clinical coordination, blurring the line between work‐related and personal use. This study could not differentiate these usage types, potentially underestimating the functional value of work‐related use or overestimating the harm of personal use.

#### 4.8.4. Cross‐Sectional Design and Temporal Ambiguity

The cross‐sectional design precludes establishing temporal precedence. Reverse causality is plausible: nurses with inherently lower caring tendencies may both phub more and report higher exhaustion, or emotionally exhausted nurses may seek smartphone escapism as a coping mechanism. Future EMA or longitudinal designs with at least two time points are warranted.

## 5. Conclusion

Based on empirical data from 512 emergency nurses at hospitals of all levels in Sichuan Province and grounded in the JD‐R model, this study found that phubbing is directly associated with lower caring behaviors and indirectly associated with lower quality of care through emotional exhaustion, with the mediation effect accounting for 43.8% of the total effect. From a nursing management perspective, phubbing should not be viewed as an individual disciplinary issue but as a maladaptive coping symptom arising from an imbalance between high job demands and low job resources. Effective management interventions must transcend “ban mentality” and adopt JD‐R‐oriented comprehensive strategies: increasing work resources through specialist empowerment and tiered mentorship, reducing environmental triggers through contextual smartphone use regulation, and restoring psychological energy through emotional exhaustion interventions and adaptive resource recovery training. Only through coordinated efforts at the organizational, leadership, and individual levels can the negative association pattern of ‘phubbing–exhaustion–low‐quality care’ be prevented to ensure the safety and care experience of emergency patients.

## Funding

This research received no specific grant from any funding agency in the public, commercial, or not‐for‐profit sectors.

## Conflicts of Interest

The authors declare no conflicts of interest.

## Data Availability

The de‐identified dataset and study materials are available from the corresponding author upon reasonable request, subject to institutional ethical approval constraints.
